# X chromosome aneuploidies and schizophrenia: association analysis and phenotypic characterization

**DOI:** 10.1111/pcn.13474

**Published:** 2022-09-24

**Authors:** Itaru Kushima, Branko Aleksic, Hiroki Kimura, Masahiro Nakatochi, Tzuyao Lo, Masashi Ikeda, Makoto Arai, Ryota Hashimoto, Shusuke Numata, Yasunobu Okamura, Taku Obara, Toshiya Inada, Norio Ozaki

**Affiliations:** ^1^ Department of Psychiatry Nagoya University Graduate School of Medicine Nagoya Japan; ^2^ Medical Genomics Center Nagoya University Hospital Nagoya Japan; ^3^ Public Health Informatics Unit, Department of Integrated Health Sciences Nagoya University Graduate School of Medicine Nagoya Japan; ^4^ Department of Psychiatry Fujita Health University School of Medicine Toyoake Japan; ^5^ Department of Psychiatry and Behavioral Sciences Tokyo Metropolitan Institute of Medical Science Tokyo Japan; ^6^ Department of Pathology of Mental Diseases National Institute of Mental Health, National Center of Neurology and Psychiatry Kodaira Japan; ^7^ Department of Psychiatry, Graduate School of Biomedical Science Tokushima University Tokushima Japan; ^8^ Advanced Research Center for Innovations in Next‐Generation Medicine Tohoku University Sendai Japan; ^9^ Tohoku Medical Megabank Organization Tohoku University Sendai Japan; ^10^ Institute for Glyco‐core Research (iGCORE), Nagoya University Nagoya Japan

**Keywords:** comparative genomic hybridization, schizophrenia, X chromosome

## Abstract

**Aim:**

The aims of the present study were: (i) to examine the association between schizophrenia (SCZ) and 47, XXY or 47, XXX in a large case–control sample; and (ii) to characterize the clinical features of patients with SCZ with these X chromosome aneuploidies.

**Methods:**

To identify 47, XXY and 47, XXX, array comparative genomic hybridization (aCGH) was performed in 3188 patients with SCZ and 3586 controls. We examined the association between 47, XXY and 47, XXX and SCZ in males and females separately using exact conditional tests to control for platform effects. Clinical data were retrospectively examined for patients with SCZ with X chromosome aneuploidies.

**Results:**

Of the analyzed samples, 3117 patients (97.8%) and 3519 controls (98.1%) passed our quality control. X chromosome aneuploidies were exclusively identified in patients: 47, XXY in seven patients (0.56%), 47, XXX in six patients (0.42%). Statistical analysis revealed a significant association between SCZ and 47, XXY (*P* = 0.028) and 47, XXX (*P* = 0.011). Phenotypic data were available from 12 patients. Treatment‐resistance to antipsychotics and manic symptoms were observed in six patients each (four with 47, XXY and two with 47, XXX for both), respectively. Statistical analysis revealed that treatment‐resistance to antipsychotics, mood stabilizer use, and manic symptoms were significantly more common in patients with 47, XXY than in male patients without pathogenic copy number variations.

**Conclusion:**

These findings indicate that both 47, XXY and 47, XXX are significantly associated with risk for SCZ. Patients with SCZ with 47, XXY may be characterized by treatment‐resistance and manic symptoms.

Klinefelter syndrome (47, XXY) and triple X syndrome (47, XXX) are characterized by the presence of an additional X chromosome in males and females, respectively, and are among the most common genetic disorders, occurring in one of 600 male and in one of 1000 female live births, respectively.^1^

Clinical characteristics of Klinefelter syndrome are heterogeneous, but they typically include tall stature, small testes, azoospermia, and symptoms related to hypogonadism. On the other hand, clinical characteristics of triple X syndrome are relatively scarce, other than tall stature. General intellectual ability for individuals with these X chromosome aneuploidies is mildly affected.^1^, ^2^ It has been suggested that individuals with Klinefelter and triple X syndrome may be at increased risk for psychiatric disorders.^3^, ^4^ Patients with Klinefelter syndrome have been reported to have almost a four times higher risk of schizophrenia (SCZ),^5^ and patients with triple X syndrome have shown a higher prevalence of psychotic disorders.^6^ Chromosome karyotype analysis performed before the era of microarray analysis showed that the rate of 47, XXY and 47, XXX was increased in patients with SCZ (0.54% and 0.63%, respectively).^7^

Despite these findings, 47, XXY and 47, XXX are not currently recognized as a major genetic risk factor for SCZ.^8^, ^9^, ^10^, ^11^, ^12^, ^13^ There are several reasons for this. First, although clinical studies of patients with Klinefelter or triple X syndrome have provided support for an association between SCZ and 47, XXY or 47, XXX,^5^, ^6^, ^14^, ^15^ evidence from case–control studies of SCZ remains limited. These studies were either small^16^ or used an inaccurate sex chromatin screening method (buccal smear).^7^ Although large‐scale case–control studies of SCZ have been recently published, further evidence for an association between SCZ and 47, XXY or 47, XXX has not been reported.^17^, ^18^, ^19^ Second, evidence for an association has come mainly from studies in Europid populations. Replication in other populations is needed to increase the confidence in the generalization of the findings. Third, the clinical features of patients with SCZ with X chromosome aneuploidies remain unclear. Such information would also be useful for optimal treatment selection and accurate prognosis prediction.

We previously performed array comparative genomic hybridization (aCGH) analysis (*n* = 4553) and found that the rate of X chromosome aneuploidies (47, XXY and 47, XXX combined) was increased (0.41%) in patients with SCZ.^20^ In the present study, we expanded the sample size (*n* = 6636) to investigate whether both 47, XXY and 47, XXX are significantly associated with risk for SCZ. We also aimed to determine the clinical features of these patients.

## Methods

### Participants

We studied 3188 patients with SCZ (1710 males and 1478 females) and 3586 psychiatrically normal controls (1654 males and 1932 females). Patients were diagnosed according to the Diagnostic and Statistical Manual of Mental Disorders, Fifth Edition (DSM‐5), criteria for SCZ. Controls were selected from the general population and had no history of mental disorders based on responses to questionnaires or self‐reporting.

The study protocol was approved by the ethics committee of Nagoya University, Fujita Health University, Tokyo Metropolitan Institute of Medical Science, National Center of Neurology and Psychiatry, Tokushima University, and Tohoku University, and it is conformed to the provisions of the Declaration of Helsinki. Written informed consent was obtained from all participants.

### aCGH

Genomic DNA was extracted from blood or saliva samples of study participants. To identify X chromosome aneuploidies, we performed aCGH using two platforms: NimbleGen 720k Whole‐Genome Tiling array (Roche NimbleGen, Madison, WI) and Agilent SurePrint G3 human CGH 400k (Agilent, Santa Clara, CA). From the aCGH data, we generated copy number variation (CNV) calls using Nexus Copy Number software v9.0 (BioDiscovery, El Segundo, CA). Briefly, the same log2 ratio thresholds for CNV calls were used for aCGH data from NimbleGen and Agilent arrays: −0.4 (loss) and 0.3 (gain).

The significance threshold to adjust the sensitivity of the segmentation algorithm was set at 1 × 10^−6^ in both arrays. Three contiguous probes were required for CNV calls in the NimbleGen and Agilent arrays. A noise‐reduction algorithm for aCGH data was used for the systematic correction of artifacts caused by GC content and fragment length.^21^ Quality control (QC) scores were calculated for each sample based on the statistical variance of the probe‐to‐probe log ratios. These QC scores showed the quality of the sample and experiment, with lower scores indicating better quality results. We excluded samples with a QC score >0.2, gender mismatch, or excessive autosomal CNV calls. After the QC, 47, XXY and 47, XXX were identified in our samples.

### Quantitative polymerase chain reaction (qPCR)

We validated X chromosome aneuploidies using qPCR. TaqMan Copy Number Assays were performed using four probes targeting different regions of the X chromosome (Hs04114669_cn, Hs00120240_cn, Hs05601664_cn, and Hs05615735_cn). In addition, to exclude the possibility of an artifact due to a sex mismatch, we performed qPCR analysis of the SRY gene on chromosome Y and confirmed the presence and absence of chromosome Y in patients with 47, XXY and 47, XXX, respectively.

### Statistical analysis

The proportions of 47, XXY and 47, XXX carriers were calculated in male and female patients, respectively. The proportions were calculated for Agilent and NimbleGen platforms and then integrated using the fixed‐effect inverted variance method with the logit transformation. The heterogeneity of the proportions was assessed by Cochran's Q statistic.

We examined the association of 47, XXY and 47, XXX with SCZ in males and females separately, using exact conditional tests to control for the effects of the two platforms. In addition, the association of both aneuploidies (47, XXY + 47, XXX) with SCZ was also evaluated using the exact conditional test to control for the two platforms. The exact test was used rather than the Mantel–Haenszel test because the exact test does not use an asymptotic approximation.

### Case presentation

We retrospectively obtained longitudinal clinical data of patients with SCZ with 47, XXY or 47, XXX from medical records. The data included developmental history, age at onset, psychiatric symptoms, duration of hospitalizations, medications and their effectiveness, and the results of brain imaging, laboratory tests, and cognitive tests. Treatment‐resistant SCZ (TRS) was defined as the absence of satisfactory clinical improvement despite the use of adequate doses of antipsychotics (total chlorpromazine [CPZ] equivalent dose of >1000 mg/day). This definition is a simplified version of the definition used in the study by Kane *et al*.^22^

### Phenotypic analysis

We tested for an association between: (i) TRS and 47, XXY/47, XXX; (ii) mood stabilizer use and 47, XXY/47, XXX; and (iii) manic symptoms and 47, XXY/47, XXX. Data for TRS and mood stabilizer use were available from 689 SCZ patients (Male: 53.3%) without pathogenic CNVs. Data for manic symptoms were available from 50 SCZ patients (Male: 42.0%) without pathogenic CNVs. Age was matched in patients with 47, XXY/47, XXX and patients without pathogenic CNVs. Pathogenic CNVs were defined as reported in our previous study.^23^

## Results

### Association analysis

Of the 3188 patients and 3586 controls analyzed with aCGH, 3117 (97.8%: 1668 males, 1449 females) and 3519 (98.1%: 1627 males, 1892 females) passed QC, respectively. We identified 47, XXY in seven male patients (0.56%; 95% confidence interval [CI] 0.27–1.17) and 47, XXX in six female patients (0.42%; 95% CI 0.19–0.92) (Supplementary Fig. S1). No significant heterogeneity was found in the proportion between the two platforms (*P* value of Q > 0.05). These aneuploidies were confirmed by qPCR (Supplementary Fig. 2). The identification of 47, XXY in one patient (patient 7) was a *de novo* event. With the exception of patient 5 (47, XXY and 16p13.11 deletion), no patients had other pathogenic CNVs (Table 1). No X chromosome aneuploidies were identified in the controls. Statistical analysis revealed that both 47, XXY and 47, XXX were significantly associated with increased risk for SCZ (47, XXY: odds ratio [OR] = ∞, *P* = 0.028; 47, XXX: OR = ∞, *P* = 0.011). Combining 47, XXY and 47, XXX showed a stronger association with SCZ (OR = ∞, *P* = 0.00030).

**Table 1:** Clinical phenotypes of seven patients with SCZ with 47, XXY

Patient	Patient 1	Patient 2	Patient 3	Patient 4	Patient 5	Patient 6	Patient 7
Genetic findings	47, XXY	47, XXY	47, XXY	47, XXY	47, XXY	47, XXY	47, XXY
					16p13.11 deletion		
Age (years)/sex	60/M	61/M	70/M	54/M	39/M	60/M	29/M
Family history of psychiatric disorders	+	−	−	−	+ (SCZ)	−	+
Developmental history	Delay in language development, peculiar behavior, poor academic performance	Poor academic performance	Unremarkable	Introverted personality	Poor rearing environment, poor academic performance, trichotillomania	Unsociable	Unremarkable
Education (years)	9	12	12	16	9	12	12
Number of children	0	0	0	0	0	0	0
Age at onset	42	24	25	30	15	28	21
Main psychiatric symptoms	Persecutory delusions, auditory hallucinations, catatonic stupor	Persecutory delusions, auditory/visual hallucinations, manic agitation	Delusions, hallucinations, manic symptoms,	Negative symptoms, catatonic stupor, agitation, mild psychotic symptoms	Grandiose delusions, auditory hallucinations	Auditory hallucinations, persecutory delusions, social withdrawal	Thought broadcasting, negative symptoms, anosognosia
Hospitalization	~20 years	~30 years	35 years	2 years	3 years	1 year	7 years
Medications prescribed at study evaluation	Risperidone, haloperidol, zotepine	Perospirone, aripiprazole, paliperidone, lithium	Levomepromazine, zotepine, carbamazepine	Quetiapine, levomepromazine	Haloperidol, perospirone, zotepine, lithium, valproate	Chlorpromazine, risperidone	Chlorpromazine, paliperidone, valproate
Treatment‐resistance	+	+	−	−	+	−	+
Intellectual disability	Possible	−	−	−	Possible	−	−
Height/weight	160‐cm/48‐kg	164‐cm/59.5‐kg	163‐cm/71‐kg	NA	166‐cm/68‐kg	165‐cm/69‐kg	187‐cm/65‐kg
Physical anomalies and illnesses	−	−	Diabetes mellitus, hyperlipidemia, obesity	Diabetes mellitus	−	Diabetes mellitus, hyperlipidemia, hepatitis C, osteomyelitis	Epilepsy, asthma
Brain imaging	Temporal atrophy, lateral ventricle enlargement, basal ganglia calcification	NA	Mild frontal atrophy, basal ganglia calcification	Low density areas in the parietal‐occipital lobe	No significant findings	NA	NA

Abbreviations: NA, not available; SCZ, schizophrenia.

### Case presentation

We obtained phenotypic data from 12 patients (seven with 47, XXY and five with 47, XXX). These data are described below and summarized in Tables 1 and 2. No patients were diagnosed as having 47, XXY/47, XXX prior to the aCGH analysis.

**Table 2:** Clinical phenotypes of five patients with SCZ with 47, XXX

Patient	Patient 8	Patient 9	Patient 10	Patient 11	Patient 12
Genetic findings	47, XXX	47, XXX	47, XXX	47, XXX	47, XXX
Age (years)/sex	76/F	53/F	39/F	35/F	19/F
Family history of psychiatric disorders	+ (SCZ)	+	−	−	+ (epilepsy, intellectual disability)
Developmental history	Unremarkable	Unremarkable	Unremarkable	Premature baby	Developmental delay
Education (years)	8	9	12	15	12
Number of children	3	0	0	0	0
Age at onset	39	28	36	33	18
Main psychiatric symptoms	Persecutory/erotomanic delusions, cognitive deterioration, agitation	Auditory hallucinations, persecutory delusions, suicide attempts, agitation	Auditory hallucinations, circumstantial thinking	Auditory hallucinations, persecutory delusions, negative symptoms, cognitive impairments	Persecutory delusions, delusions of observation, auditory hallucinations, thought broadcasting
Hospitalization	~30 years	> 30 years	−	< 1 year	< 1 year
Medications prescribed at study evaluation	Perospirone	Olanzapine, zotepine, carbamazepine	Olanzapine	Olanzapine	Blonanserin, valproate, carbamazepine
Treatment‐resistance	+	+	−	−	−
Intellectual disability	−	−	−	−	Possible
Height/weight	150‐cm/38.7‐kg	NA	NA	155‐cm/49‐kg	156‐cm/60‐kg
Physical anomalies and illnesses	Exotropia	Atrioventricular block	−	−	Epilepsy
Brain imaging	Temporal‐occipital atrophy, basal ganglia calcification	NA	NA	No significant findings	NA

Abbreviations: NA, not available; SCZ, schizophrenia.

### Patients 1–7: 47, XXY

Patient 1 was a 60‐year‐old male with TRS. He had a family history of a psychiatric disorder (his brother). As a child, he had possible intellectual disability and showed delayed language development and peculiar behavior. In school, his academic performance was poor. After graduating from junior high school, he worked in factories for around 25 years. At the age of 42 years, he developed persecutory delusions and auditory hallucinations. His psychotic symptoms did not improve much with antipsychotic treatment and he was hospitalized in a psychiatric hospital for about 20 years. At the time of this evaluation, delusions and hallucinations persisted despite high doses of antipsychotics (risperidone, haloperidol, zotepine: CPZ equivalent dose >1500 mg/day). Every 2 months, his psychotic symptoms worsened and he fell into a catatonic stupor. Brain computed tomography (CT) revealed temporal atrophy, lateral ventricle enlargement, and basal ganglia calcification.

Patient 2 was a 61‐year‐old male with TRS. His early developmental history was unremarkable, but his academic performance was poor in school. After graduating from junior high school, he worked for several companies until the onset of SCZ. At the age of 24 years, he developed persecutory delusions and auditory and visual hallucinations. Even after treatment initiation, he experienced recurrent psychotic episodes with manic agitation and had to be hospitalized 12 times (in total, over about 30 years). His medications at the time of the study evaluation included antipsychotics (perospirone, aripiprazole, paliperidone: CPZ equivalent dose, 1200 mg/day) and a mood stabilizer (lithium 400 mg/day). His test results were not available. He had no remarkable medical conditions.

Patient 3, a 70‐year‐old male, had an unremarkable developmental history. His grades were average in school. He started to work after high school, but only worked for a short time and moved from job to job. At the age of 25 years, he had delusions and hallucinations with manic features (elevated mood and agitation). He responded to antipsychotic treatment in hospital for his psychotic symptoms, but then had repeated relapses of psychosis with manic features. He was hospitalized 14 times (in total, over 35 years). During these long‐term hospitalizations, he was treated with antipsychotics (levomepromazine and zotepine) and a mood stabilizer (carbamazepine). Over time, negative symptoms such as avolition and social withdrawal became predominant. He had obesity, diabetes mellitus, and hyperlipidemia. Brain CT showed mild frontal atrophy and basal ganglia calcification.

Patient 4, a 54‐year‐old male, had an unremarkable developmental history. His academic performance was average in school. After graduating from university, he worked for a company. At the age of 30 years, he began to suffer from insomnia and to avoid other people, becoming withdrawn. Disorganized behavior with agitation and mild psychotic symptoms were also observed. His symptoms improved after inpatient treatment and he stabilized with antipsychotics (quetiapine and levomepromazine) over 20 years. At the age of 56 years, he began to complain of somatic symptoms. His psychiatric symptoms eventually worsened and he fell into a catatonic stupor. He had diabetes mellitus. Electroencephalography was normal. Brain CT showed low density areas in the parietal‐occipital lobe.

Patient 5 was a 39‐year‐old male with TRS. He had not only 47, XXY, but also 16p13.11 deletion. His family history was positive for SCZ (his father). He grew up in a poor rearing environment. In school, his academic performance was poor. After graduating from junior high school, he became withdrawn and had trichotillomania. He also committed violence toward his family. At the age of 15 years, he developed SCZ but refused to undergo treatment for over 10 years. At the age of 28 years, he had prominent grandiose delusions and was hospitalized for a long time. At the time of this evaluation, he was being treated with high doses of antipsychotics (haloperidol, perospirone, zotepine: CPZ equivalent dose >1200 mg/day) and mood stabilizers (lithium 600 mg/day and valproate 600 mg/day), but his delusions did not improve much. His full‐scale intelligence quotient (IQ) was 52, which may reflect intellectual disability and/or cognitive deterioration after the onset of SCZ. He did not have any medical conditions. Brain CT revealed no significant findings.

Patient 6 was a 60‐year‐old male with no family history of psychiatric disorders. As a child, he was unsociable but did well in school. After graduating from junior high school, he worked part‐time. At the age of 28 years, he developed auditory hallucinations and began to commit violence toward his family. Thereafter, he continued to be socially withdrawn and remained untreated for over 10 years. In his early 40s, still suffering from persecutory delusions, he was hospitalized for the first time. His conditions improved with modified electroconvulsive therapy and antipsychotic medications. After discharge, he was able to live his life. His medical history included diabetes mellites, hyperlipidemia, hepatitis C, and osteomyelitis.

Patient 7 was a 29‐year‐old male with TRS. His family history was positive for psychiatric disorder. He did not show any delays in motor or speech development. After graduating from a vocational school, he gradually became withdrawn. He showed thought broadcasting, negative symptoms (e.g. flattened expressions), and anosognosia. His conditions were resistant to antipsychotics. His full‐scale IQ was 64. He had a history of epilepsy and asthma. He also had tall stature (height: 187‐cm, weight: 65‐kg).

### Patients 8–12: 47, XXX

Patient 8 was a 76‐year‐old female with TRS. Her family history was positive for SCZ (her sibling and child). She had an unremarkable developmental history. After finishing elementary school, she worked for years and got married. At the age of 39 years, she had persecutory and erotomanic delusions and became agitated. Her psychotic symptoms were refractory to antipsychotics, and she had recurrent psychotic episodes, resulting in multiple hospitalizations (~30 years). Her cognitive functioning gradually deteriorated, and her behavior and speech eventually became completely disorganized. Brain CT revealed calcification at the basal ganglia and prominent atrophy at the temporal and occipital cortex. She also had exotropia.

Patient 9 was a 53‐year‐old female with TRS. Her developmental history was unremarkable. After finishing junior high school, she worked in a factory. In her mid‐20s, she made multiple suicidal attempts with high intentionality. At the age of 28 years, she developed auditory and persecutory hallucinations. Despite antipsychotic treatment, her psychotic symptoms persisted, accompanied by suicide attempts and violent behavior. She was then admitted to a psychiatric hospital for over 30 years. At the time of this evaluation, she was receiving high doses of antipsychotics (olanzapine and zotepine: CPZ equivalent dose >1100 mg/day) and a mood stabilizer (carbamazepine 500 mg/day), but her psychotic symptoms and agitations were prominent. She did not have any medical conditions except for atrioventricular block.

Patient 10 was a 39‐year‐old female with no family history of psychiatric disorders. Her developmental history was unremarkable. After finishing high school, she worked as a clerk and got married. At the age of 36 years, she had auditory hallucinations and thought disorder (i.e. circumstantial thinking). These symptoms rapidly resolved with small doses of olanzapine. She had no medical conditions. At the time of this evaluation, her social functioning was good and she was working as a clerk.

Patient 11 was a 35‐year‐old female with no family history of psychiatric disorders. She was born prematurely, but had an otherwise unremarkable developmental history. In school, her grades were average. After graduating from junior college, she helped out with the family business. At the age of 33 years, she developed auditory and persecutory delusions. Her psychotic symptoms improved with olanzapine and were subsequently controlled with small doses of olanzapine. At the time of this evaluation, negative symptoms and cognitive impairments were prominent. She had no comorbidities/underlying medical conditions or significant findings on brain MRI.

Patient 12 was a 19‐year‐old female with possible intellectual disability. She had a family history of intellectual disability and epilepsy. At the age of 4 years, she developed epilepsy and received treatment for 4 years. She was noted to have developmental delay at the age of 6 years, but entered a regular class and went on to junior high school. Her full‐scale IQ was 74 at the age of 12 years. At the age of 18 years, she developed persecutory delusions, delusions of observation, auditory hallucinations, and thought broadcasting. She responded to antipsychotic treatment and was able to attend day care regularly. However, she still needed support from her parents to lead her daily life.

### Phenotypic analysis

TRS was significantly more common in patients with 47, XXY than in male patients without pathogenic CNVs (57.1% *vs*. 17.7%, respectively; OR = 6.2, *P* = 0.024). No significant difference in the TRS rate between patients with 47, XXX and female patients without pathogenic CNVs was observed (*P* > 0.1).

The use of mood stabilizers (lithium, carbamazepine, and valproate) was significantly more common in patients with 47, XXY than in male patients without pathogenic CNVs (57.1% *vs*. 17.4%, OR = 6.3, *P* = 0.023). No significant difference in the rate of mood stabilizer use was found between patients with 47, XXX and female patients without pathogenic CNVs (*P* > 0.1).

Manic symptoms were significantly more common in patients with 47, XXY than in male patients without pathogenic CNVs (57.1% *vs*. 14.3%, respectively; OR = 8.0, *P* = 0.043). No significant difference in the rate of manic symptoms between patients with 47, XXX and female patients without pathogenic CNVs was observed (*P* > 0.05).

## Discussion

In the present study, we found that both 47, XXY and 47, XXX are significantly associated with risk for SCZ. The frequencies of 47, XXY (0.56%) and 47, XXX (0.42%) in patients with SCZ were also high relative to those in the Japanese general population (0.07% and 0%, respectively, unpublished data in^24^).

Consistent with their low diagnosis rates,^25^ no patients were diagnosed as 47, XXY/47, XXX prior to the aCGH analysis. The following physical characteristics of Klinefelter syndrome were observed in at least one patient with 47, XXY: tall stature, diabetes mellitus, and epilepsy. However, as their specificity is not high, it may be difficult to suspect Klinefelter syndrome from these characteristics. Intellectual ability for individuals with 47, XXY/47, XXX is reported to be mildly affected.^1^, ^2^ Consistent with this, three patients had possible intellectual disability.

The phenotypic analysis also revealed that patients with SCZ with 47, XXY were more likely to have TRS. A few case reports of patients with SCZ with 47, XXY who showed resistance to antipsychotic medications have been published.^26^, ^27^ It has been reported that patients with SCZ have a higher frequency of pathogenic CNVs and that patients with pathogenic CNVs are more likely to be resistant to treatment.^28^, ^29^ Therefore, CNV analysis may be recommended as a clinical diagnostic test, especially in patients with TRS. Patients with 47, XXY were also more likely to have manic symptoms. The rate of mood stabilizer use was significantly higher in patients with SCZ with 47, XXY. Previous studies have reported a higher prevalence of bipolar disorder in patients with 47, XXY.^3^, ^5^ One patient (patient 5) with 47, XXY and 16p13.11 deletion had the earliest age at onset. The 16p13.11 deletion is associated with risk for intellectual disability and epilepsy.^30^ This is consistent with the finding that multiple pathogenic variants can lead to a severe clinical presentation.^31^

Although the mechanism by which X chromosome aneuploidies cause SCZ remains unclear, there are candidate risk genes for SCZ on the X chromosome (e.g. *MAOA*, *MAOB*, *MECP2*, *NLGN4X*).^4^, ^8^ Therefore, copy number gains of these genes may be involved in the development of SCZ in patients with X chromosome aneuploidies.

For SCZ, the finding of a specific etiology to which the psychiatric symptoms can be attributed precludes the diagnosis of SCZ (i.e. ‘another medical condition’ in criterion E for SCZ in DSM‐5). Therefore, some might argue that patients with Klinefelter syndrome or triple X syndrome should not be given a diagnosis of SCZ. The medical conditions most commonly associated with psychosis include untreated endocrine and metabolic disorders, autoimmune disorders, or temporal lobe epilepsy. On the other hand, genetic syndromes (e.g. 22q11.2 deletion syndrome, 3q29 deletion syndrome) strongly associated with ‘SCZ’ are not mentioned in the section of ‘psychotic disorder due to another medical condition’ in DSM‐5.^32^, ^33^ The reasons for this are as follows. The 22q11.2 deletion syndrome and 3q29 deletion syndrome are defined by the presence of genetic variants (i.e. 22q11.2 deletion, 3q29 deletion). These variants change the copy number of multiple genes related to brain or other organ development, and are thought to cause impaired development of brain and other organs (e.g. heart, kidney). These impairments independently increase the risk for SCZ and medical conditions. In other words, psychosis in patients with these syndromes is thought to be due to neurodevelopmental abnormalities caused by the genetic variants, rather than to the effect of the medical conditions of these syndromes (e.g. congenital heart or renal diseases). In addition, the details of neurodevelopmental abnormalities associated with these variants are unclear. Therefore, we think that genetic syndromes (not only 22q11.2 deletion syndrome and 3q29 deletion syndrome, but also Klinefelter syndrome and triple X syndrome) do not meet the exclusion criteria for SCZ.

This diagnostic issue is also discussed from a different perspective by Vorstman *et al*.^34^ As a complete description of the underlying pathological processes is not possible for most psychiatric disorders, psychiatric diagnosis in DSM‐5 relies on clinical signs and symptoms. Therefore, most psychiatric diagnoses proposed by the DSM‐5 remain naive to any established etiology. However, as rare variants with large effect size (e.g. 22q11.2 deletion, 3q29 deletion) have been identified, this has created a problematic situation of two co‐existing isolated diagnostic approaches: one descriptive (in psychiatry) and the other etiological (in genetics). As stated by Vorstman *et al*., such confusion is due to the lack of clear guidance regarding such genetic etiologies in the DSM‐5. Exceptionally, in ASD, DSM‐5 allows for maintaining the foundation of the symptom‐based diagnostic criteria, while adding genetic etiology as a specifier to the diagnosis. It is proposed that such approach in ASD should be adopted in the diagnosis of SCZ.^34^

In fact, psychiatrists have embraced that in patients with SCZ and 22q11.2 deletion, the former is caused by the latter, and a diagnosis of SCZ is most accurate, despite the presence of 22q11.2 deletion syndrome. Previous CNV studies also reported that 22q11.2 deletion syndrome and other genetic syndromes (3q29 deletion syndrome, 16p11.2 duplication syndrome, 15q11‐q13 duplication syndrome) are risk factors for SCZ.^35^, ^36^, ^37^ Furthermore, review and original papers also state that patients with Klinefelter or triple X syndrome have a higher risk for SCZ.^1^, ^5^, ^14^ In light of these situation, we believe that patients with SCZ‐like psychosis and X chromosome aneuploidies should not be excluded from SCZ.

The present study has several strengths compared with previous studies. First, to our knowledge, this is the largest single case–control study to examine the association between X chromosome aneuploidies and SCZ. Second, a reliable method (aCGH) was used to detect X chromosome aneuploidies, which were further confirmed by independent methods (qPCR). This is in contrast to previous studies using the inaccurate method of buccal smear analysis.^7^ Third, we identified the clinical characteristics of patients with SCZ with 47, XXY, which may contribute to better treatment selection and prognosis prediction. On the other hand, our study also has limitations. First, aCGH is very accurate for detecting sex chromosome aneuploidies, but not for discriminating mosaic from non‐mosaic events. Second, as we obtained phenotypic information retrospectively, our study was limited in terms of the phenotypic details.

In conclusion, the present findings suggest that both 47, XXY and 47, XXX are significantly associated with an increased risk for SCZ. In addition, patients with SCZ with 47, XXY may be characterized by TRS and manic symptoms.

## Disclosure statement

B.A., H.K., M.N., T.L., M.A., R.H., S.N., Y.O., T.O., and T.I. declare no conflict of interest. I.K. has received research grants from the SENSHIN Medical Research Foundation and Uehara Memorial Foundation. M.I. has received research grants from the Japan Agency for Medical Research and Development (AMED). N.O. has received research support or speakers' honoraria from, or has served as a consultant to, Sumitomo Dainippon, Eisai, Otsuka, KAITEKI, Mitsubishi Tanabe, Shionogi, Eli Lilly, Mochida, DAIICHI SANKYO, Nihon Medi‐Physics, Takeda, Meiji Seika Pharma, EA Pharma, Pfizer, MSD, Lundbeck Japan, and Taisho Pharma, outside the submitted work.

## Author contributions

I.K. designed the study. I.K., B.A., and T.L. performed the aCGH and/or validation experiments. I.K. and M.N. analyzed the data. I.K., H.K., M.I., M.A., R.H., S.N., Y.O., T.O., T.I., and N.O. recruited the participants and/or collected DNA samples or phenotype data. I.K. wrote the first draft of the manuscript, and the other authors commented on and refined the subsequent versions. All authors carefully read the manuscript and approved the final version for submission.

## Supporting information


**Figure S1** Results of the aCGH for X chromosome in SCZ patients with 47, XXY or 47, XXX
The figures show aCGH data of X chromosome in Patients 1–13. The vertical axis indicates log2 ratio of copy number changes. The results show copy number gain (duplication) of the X chromosome in these patients.
**Figure S2**. Results of the TaqMan Copy Number Assays in SCZ patients with 47, XXY or 47, XXX
We validated 47, XXY in Patients 1–7 and 47, XXX in Patients 8–13 using TaqMan Copy Number Assays. The assays were performed using four probes targeting different regions of the X chromosome (Hs04114669_cn, Hs00120240_cn, Hs05601664_cn, and Hs05615735_cn). Bars indicate copy numbers of X chromosome predicted by TaqMan copy number assays. The results show copy number of two in patients with 47, XXY and copy number of three in patients with 47, XXX.
