# Supplementary material for: X chromosome aneuploidies and schizophrenia: association analysis and phenotypic characterization
Source: Psychiatry Clin Neurosci. 2022 Sep 24;76(12):667–73. doi: 10.1111/pcn.13474 (PMC10086948; doi:10.1111/pcn.13474)
Supplement: Supplementary file 1 — Figure S1 Results of the aCGH for X chromosome in SCZ patients with 47, XXY or 47, XXX The figures show aCGH data of X chromosome in Patients 1–13. The vertical axis indicates log2 ratio of copy number changes. The results show copy number gain (duplication) of the X chromosome in these patients. Figure S2. Results of the TaqMan Copy Number Assays in SCZ patients with 47, XXY or 47, XXX We validated 47, XXY in Patients 1–7 and 47, XXX in Patients 8–13 using TaqMan Copy Number Assays. The assays were performed using four probes targeting different regions of the X chromosome (Hs04114669_cn, Hs00120240_cn, Hs05601664_cn, and Hs05615735_cn). Bars indicate copy numbers of X chromosome predicted by TaqMan copy number assays. The results show copy number of two in patients with 47, XXY and copy number of three in patients with 47, XXX. [file PCN-76-667-s001.docx]

**X chromosome aneuploidies and schizophrenia: association analysis and phenotypic characterization**

Running title: Schizophrenia and 47,XXY/47,XXX

Itaru Kushima^1,2,*^, Branko Aleksic^1^, Hiroki Kimura^1^, Masahiro Nakatochi^3^, Tzuyao Lo^1^, Masashi Ikeda^4^, Makoto Arai^5^, Ryota Hashimoto^6^, Shusuke Numata^7^, Yasunobu Okamura^8,9^, Taku Obara^9^, Toshiya Inada^1^, Norio Ozaki^1,10^

1. Department of Psychiatry, Nagoya University Graduate School of Medicine
2. Medical Genomics Center, Nagoya University Hospital
3. Public Health Informatics Unit, Department of Integrated Health Sciences, Nagoya University Graduate School of Medicine
4. Department of Psychiatry, Fujita Health University School of Medicine
5. Department of Psychiatry and Behavioral Sciences, Tokyo Metropolitan Institute of Medical Science
6. Department of Pathology of Mental Diseases, National Institute of Mental Health, National Center of Neurology and Psychiatry
7. Department of Psychiatry, Graduate School of Biomedical Science, Tokushima University
8. Advanced Research Center for Innovations in Next-Generation Medicine, Tohoku University
9. Tohoku Medical Megabank Organization, Tohoku University
10. Institute for Glyco-core Research (iGCORE), Nagoya University

**Supplementary Figures**

**Supplementary Figure 1.** Results of the aCGH for X chromosome in SCZ patients with 47,XXY or 47,XXX


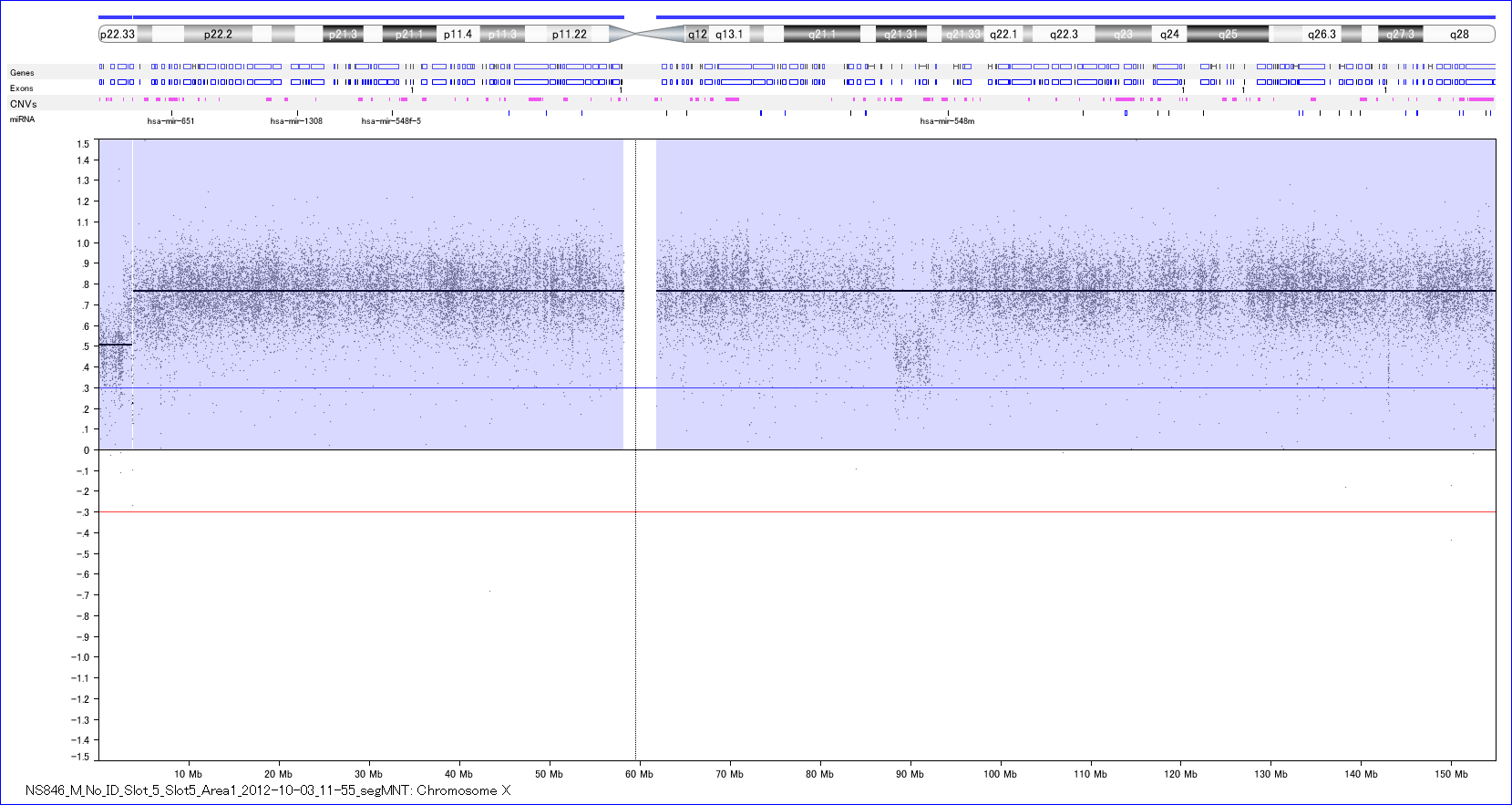


**Patient 3**


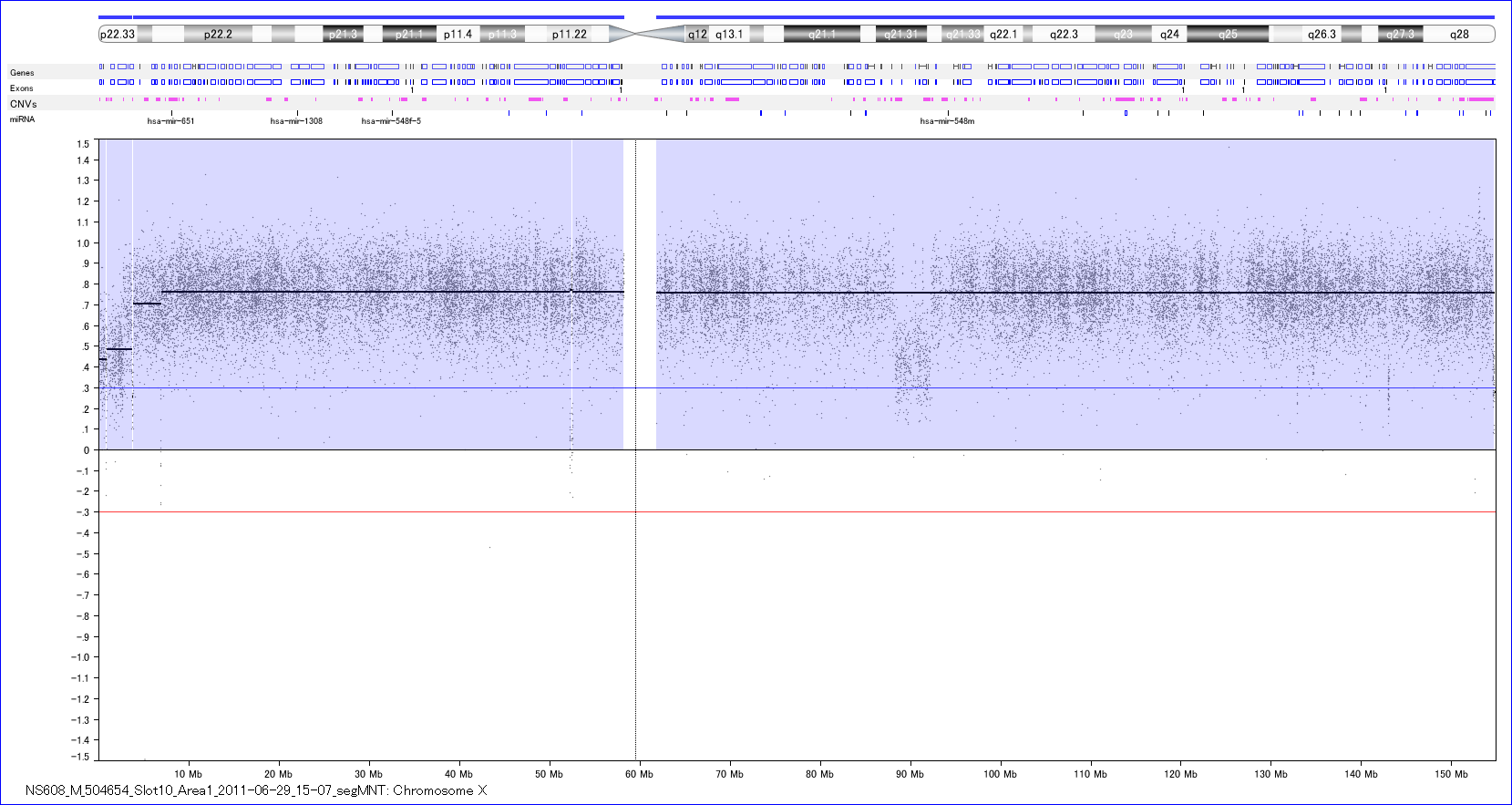


**Patient 2**


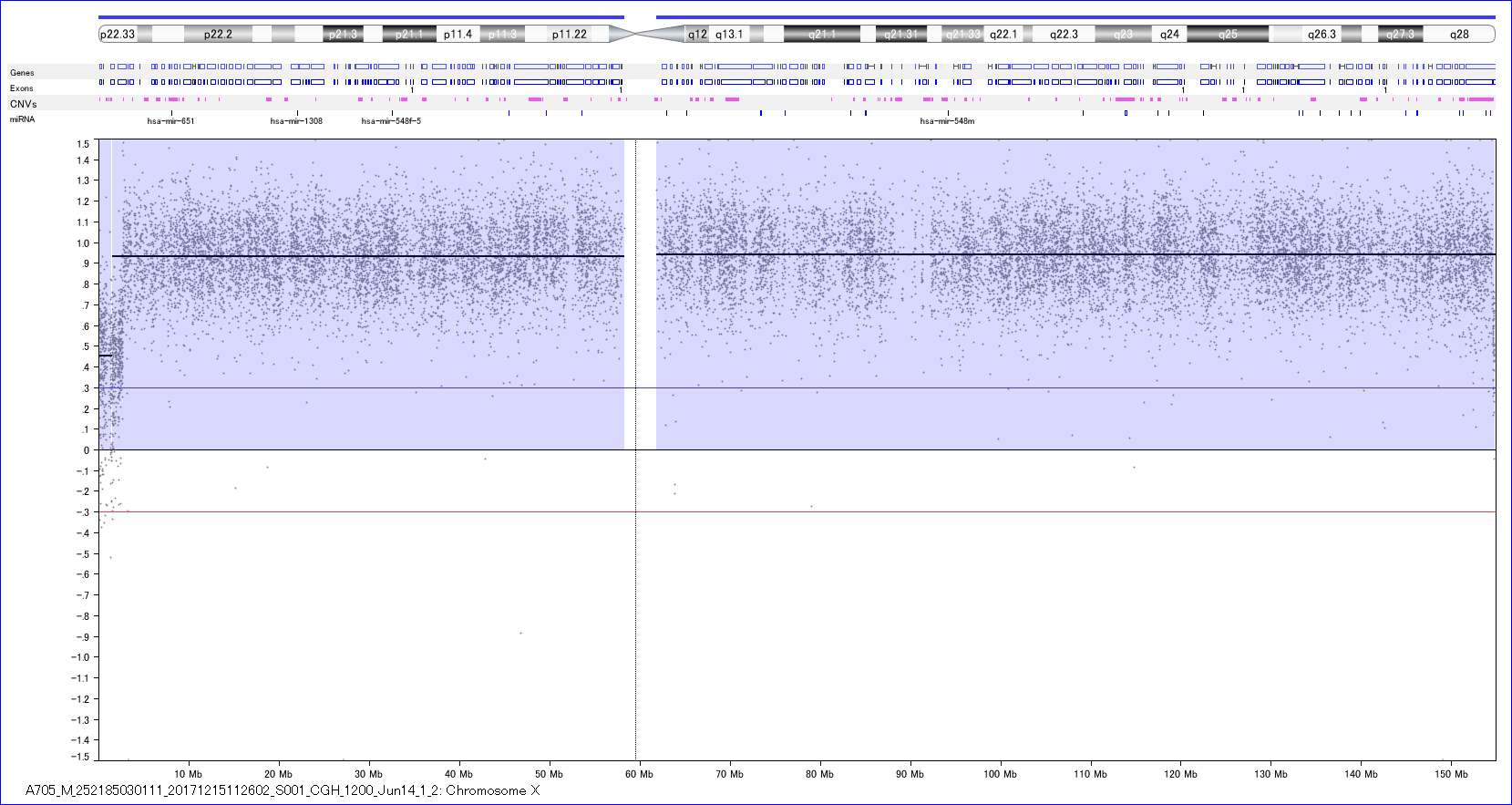


**Patient 4**


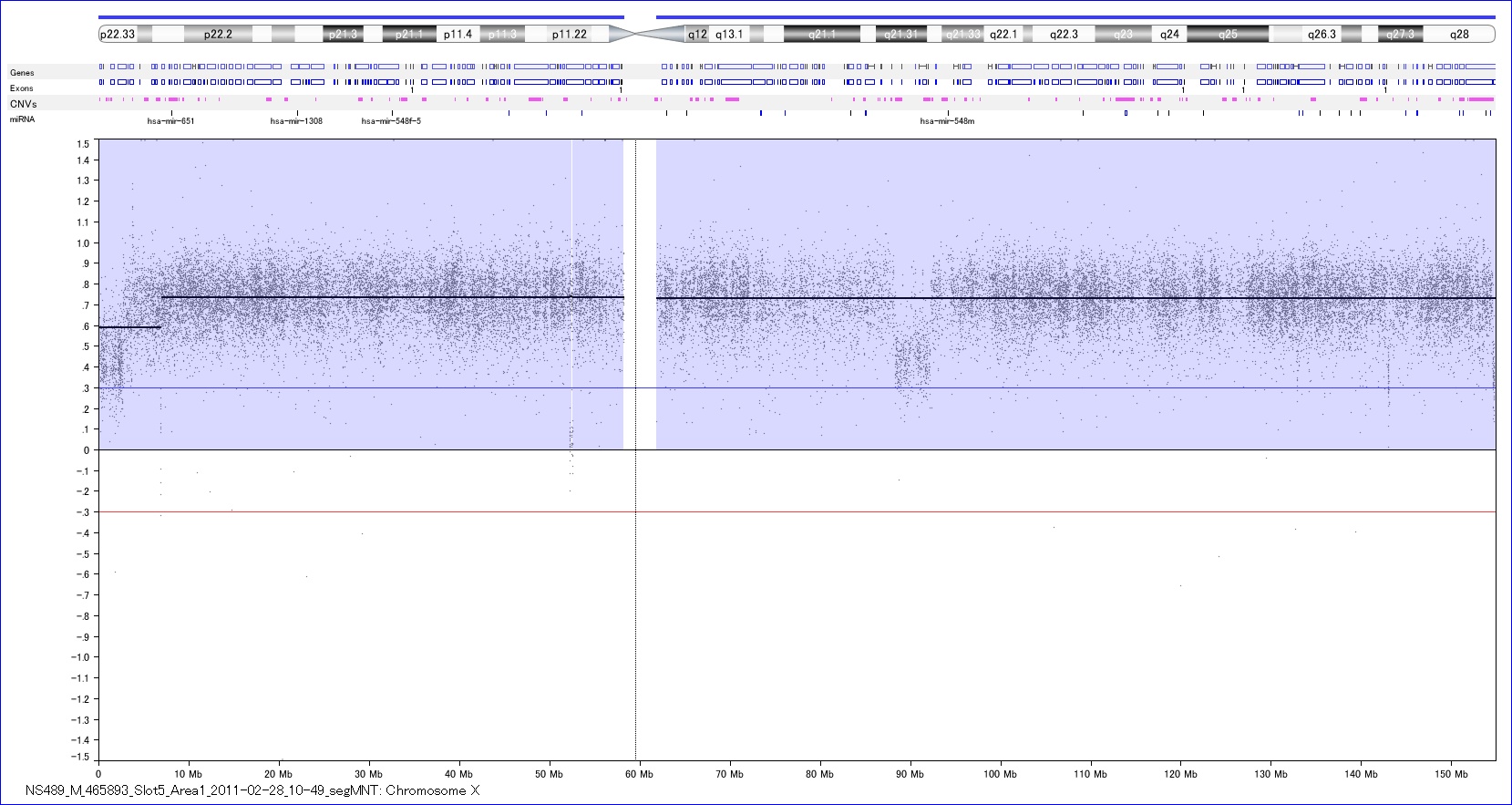


**Patient 1**


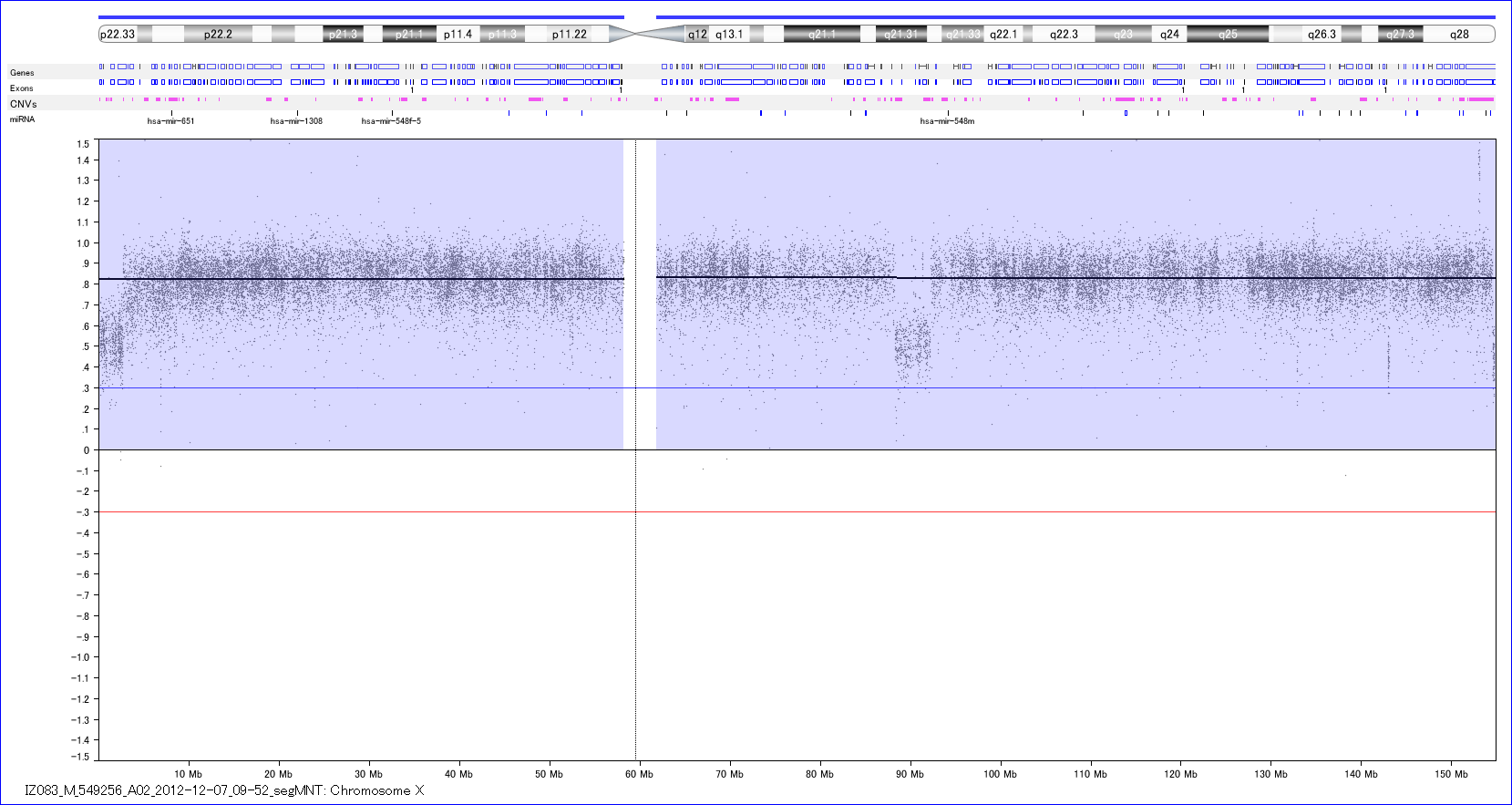


**Patient 5**


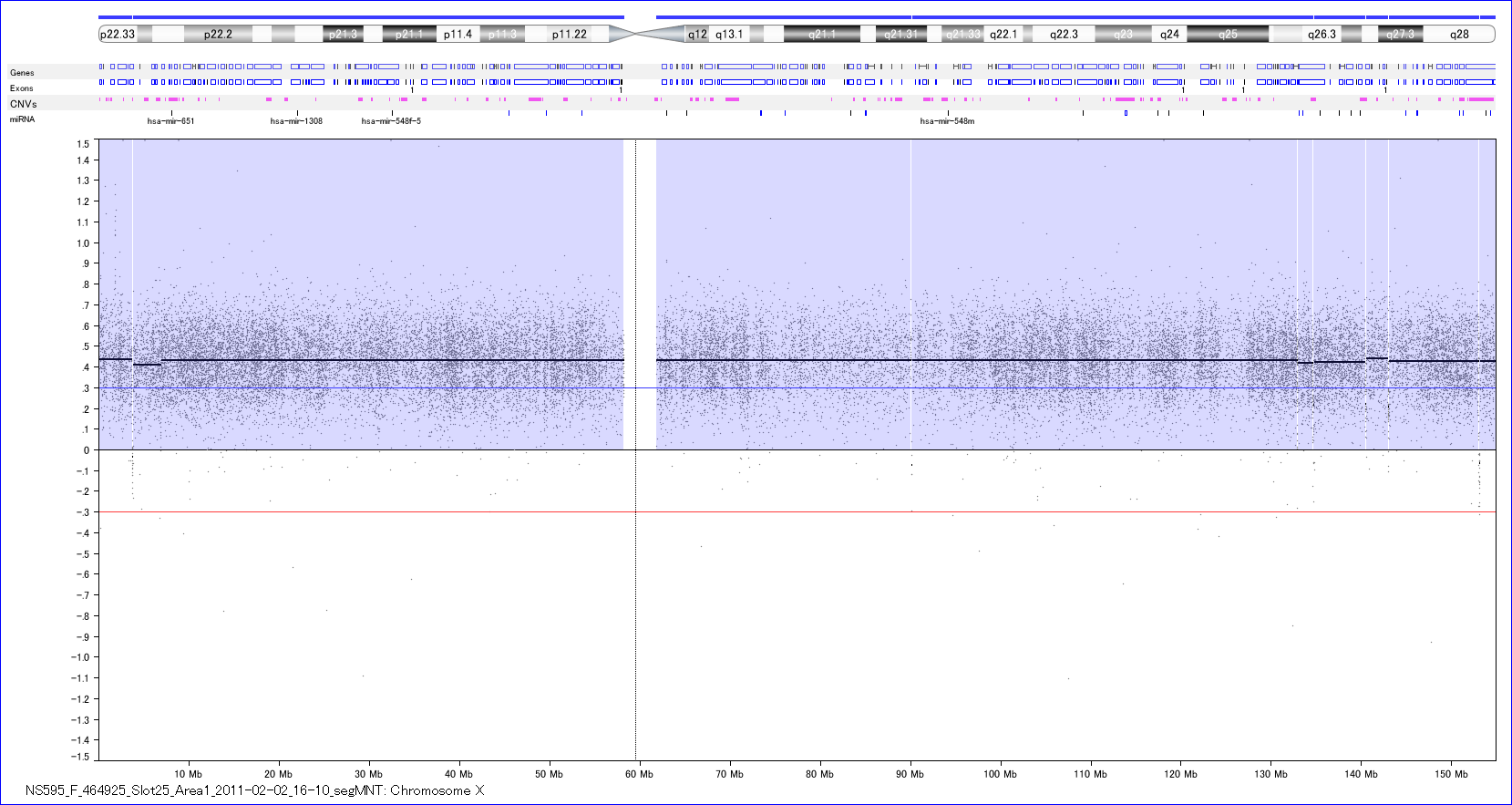


**Patient 8**


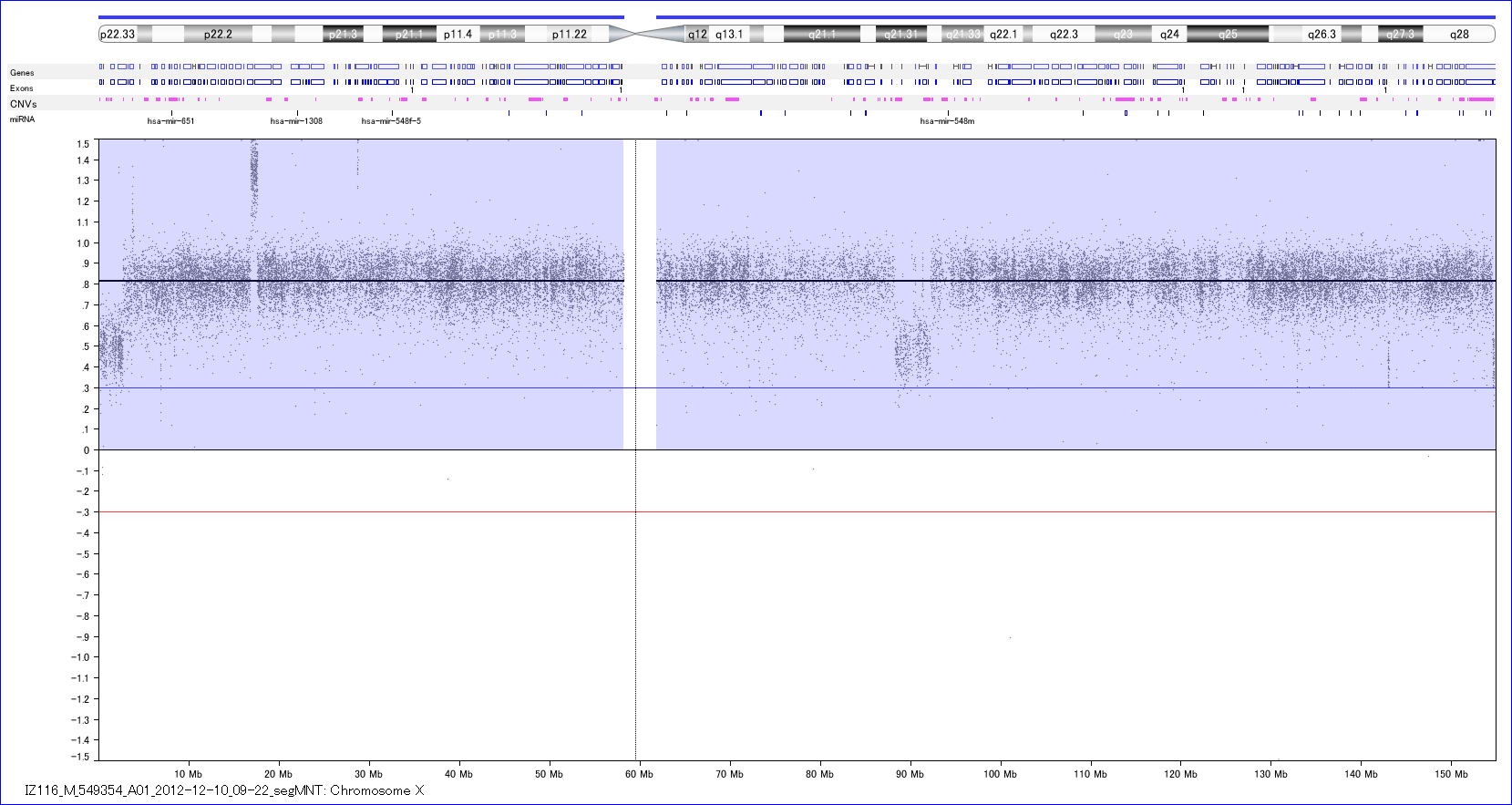


**Patient 6**


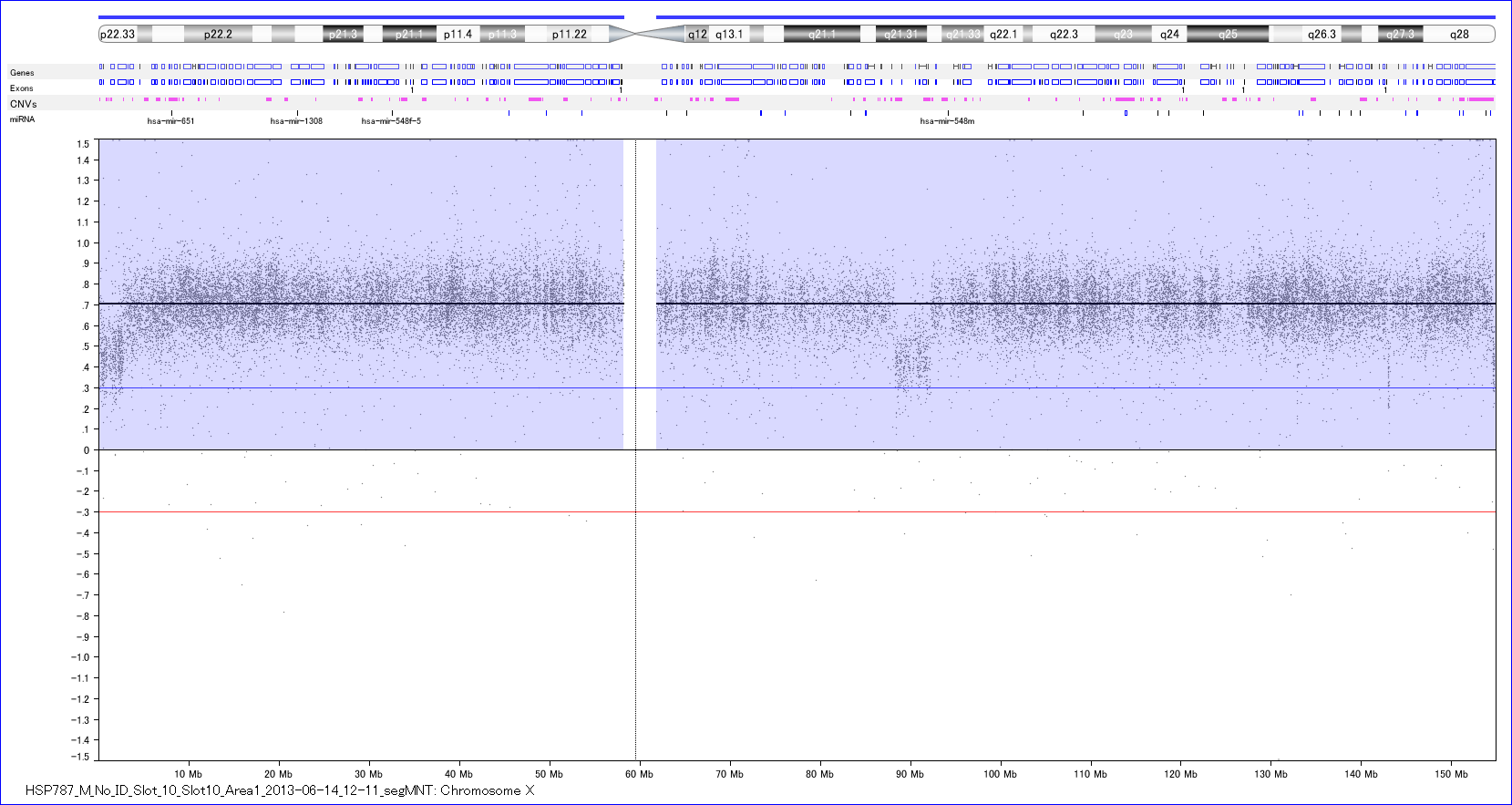


**Patient 7**


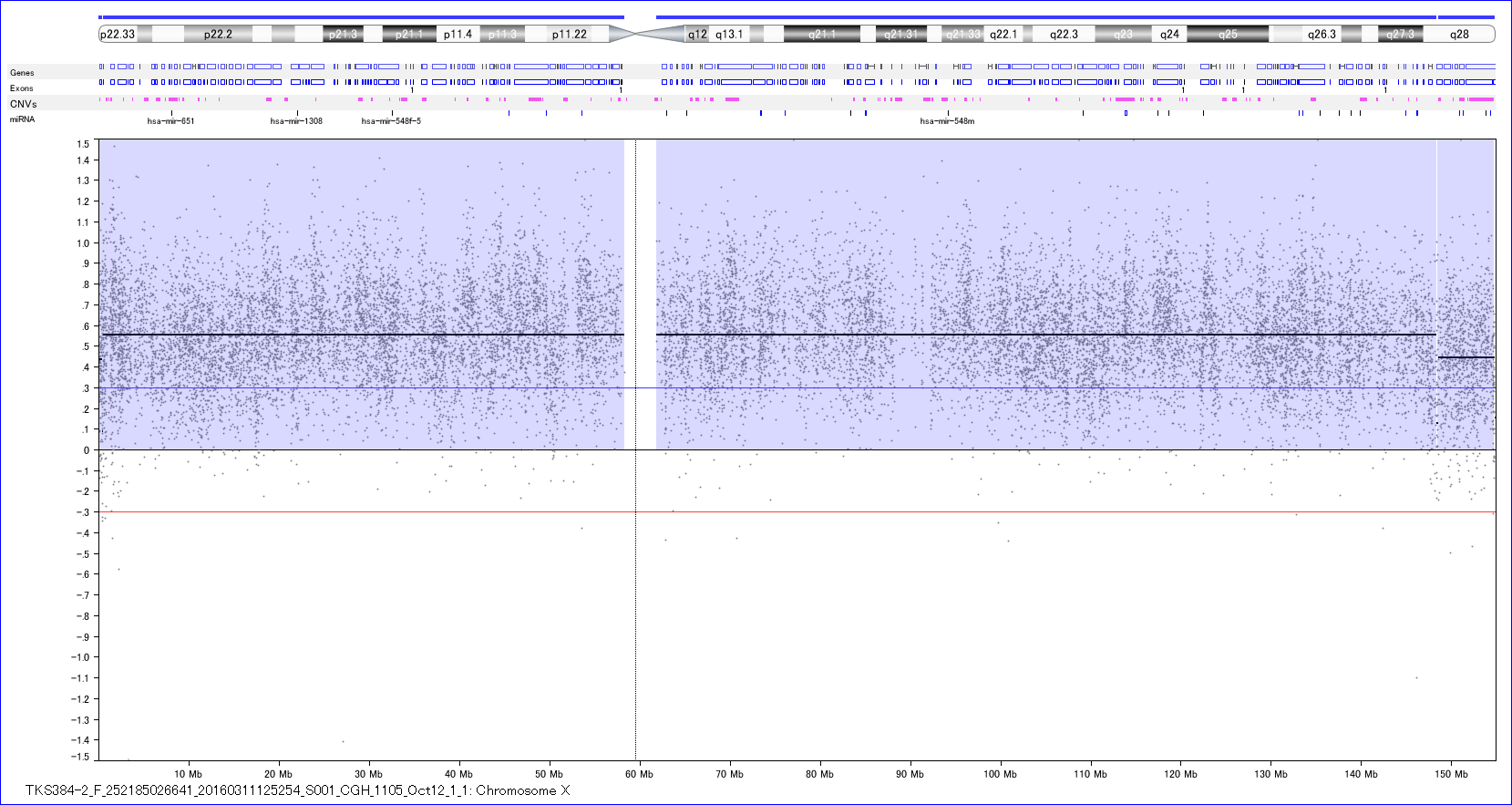


**Patient 11**


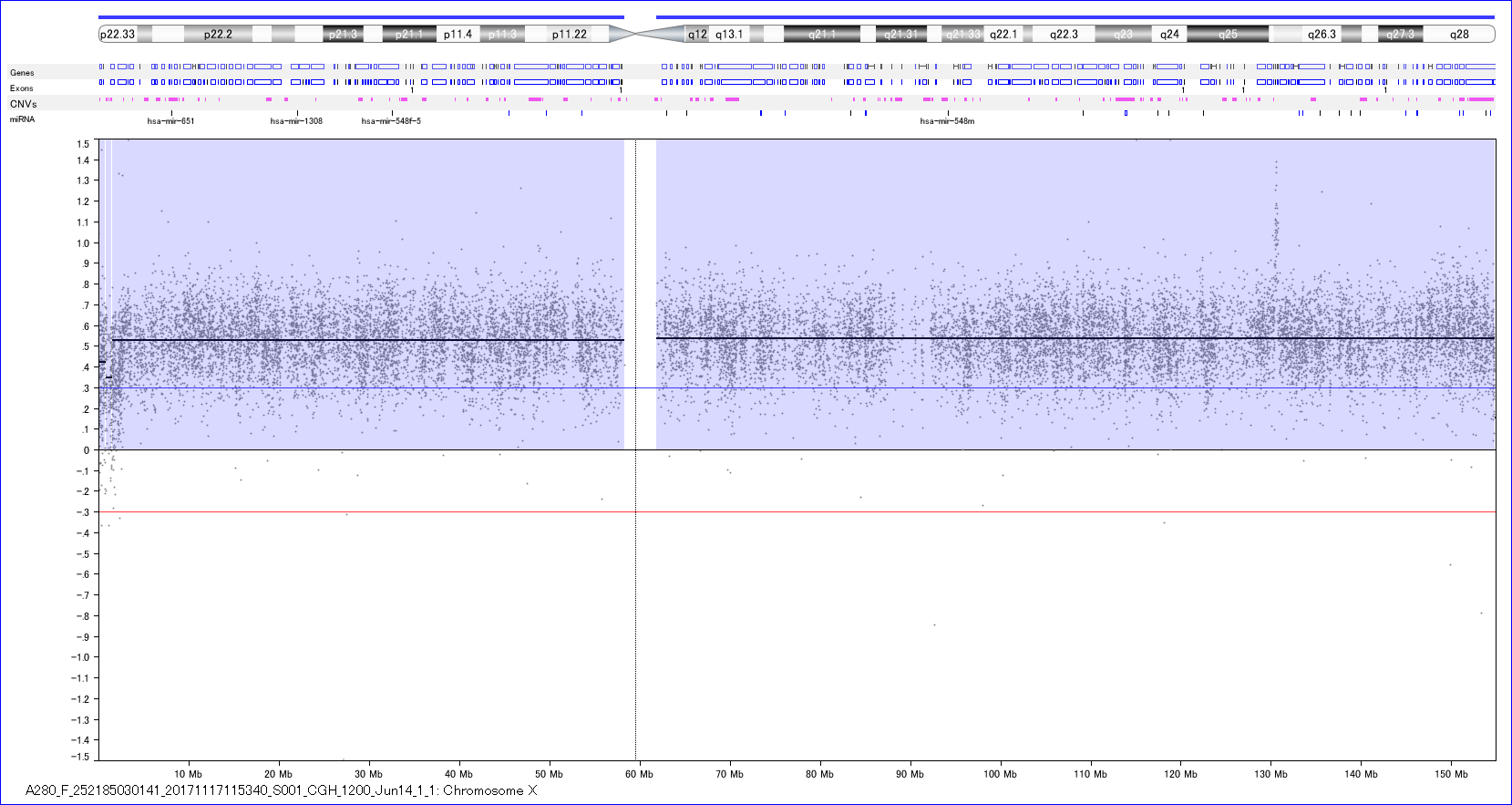


**Patient 9**


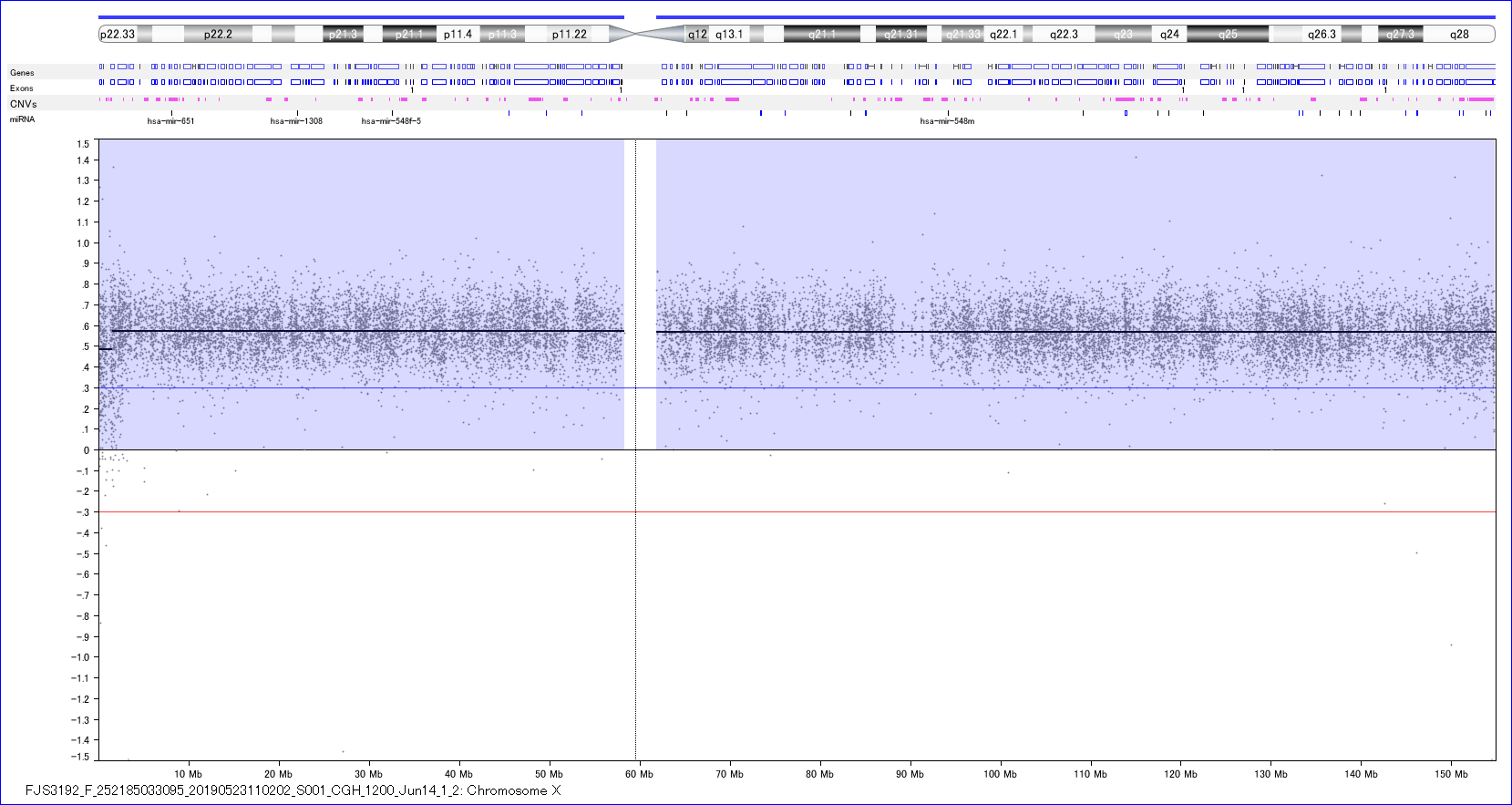


**Patient 12**


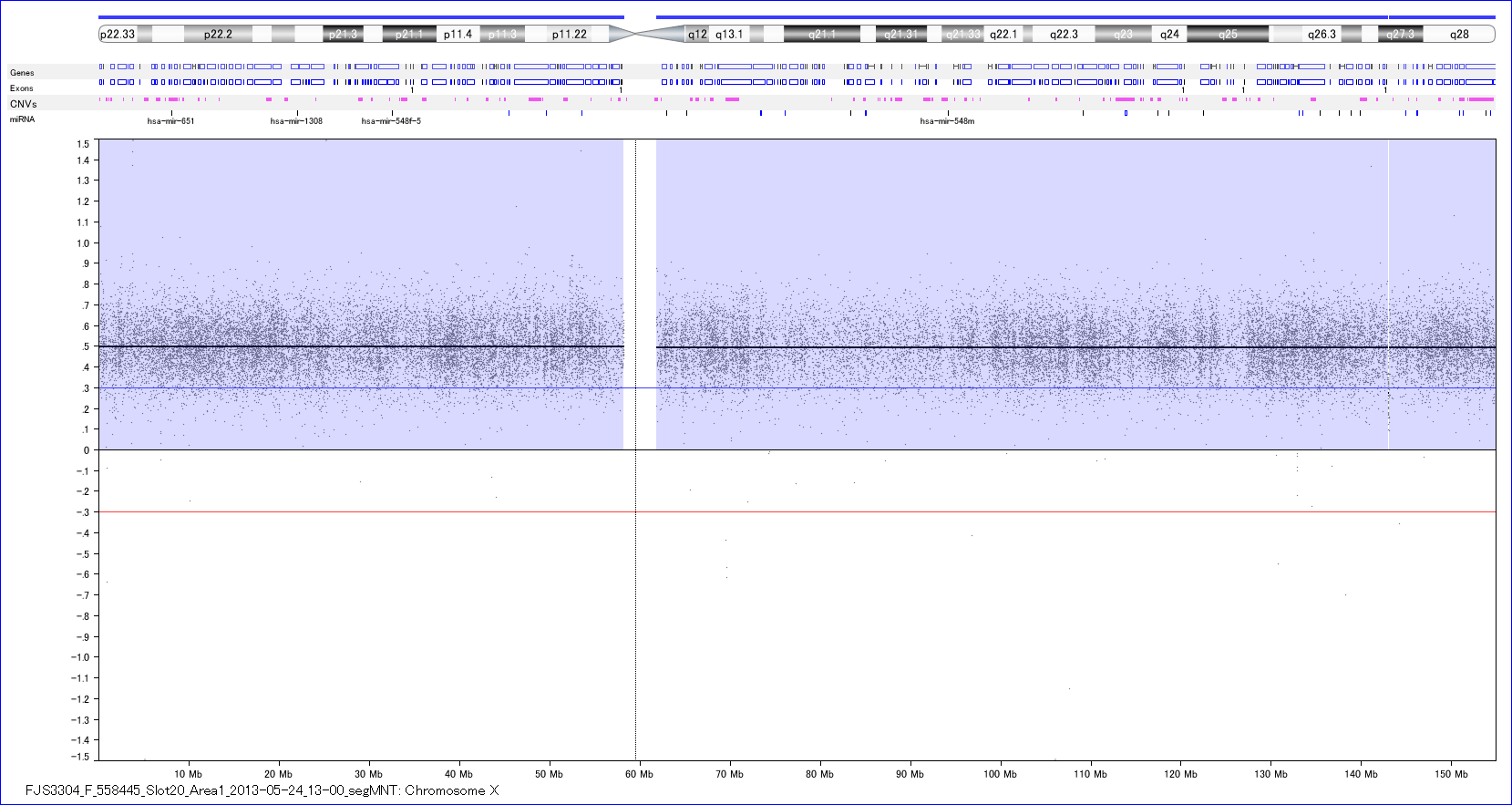


**Patient 10**


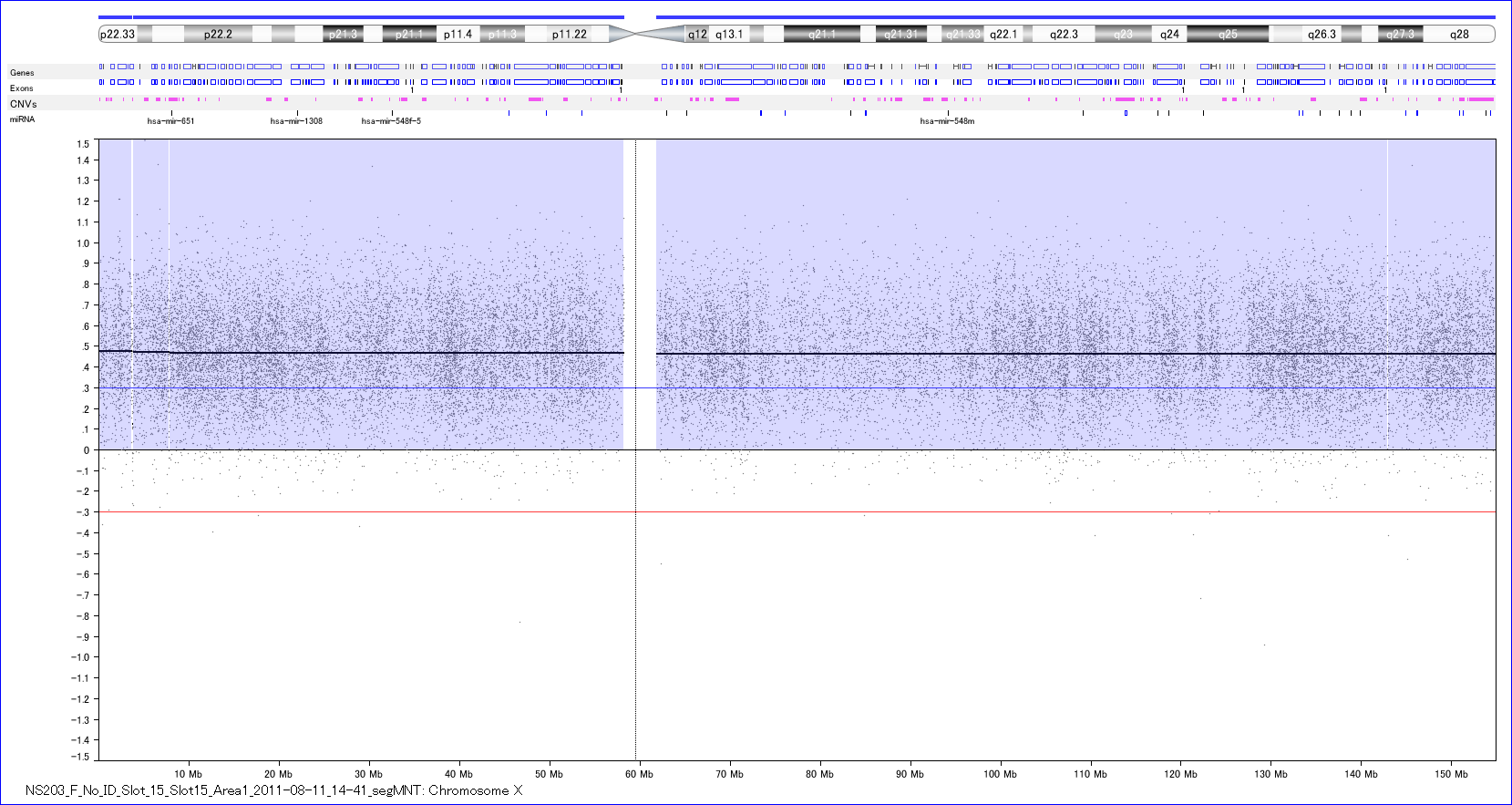


**Patient 13**

The figures show aCGH data of X chromosome in Patients 1–13. The vertical axis indicates log2 ratio of copy number changes. The results show copy number gain (duplication) of the X chromosome in these patients.

**Supplementary Figure 2**. Results of the TaqMan Copy Number Assays in SCZ patients with 47,XXY or 47,XXX


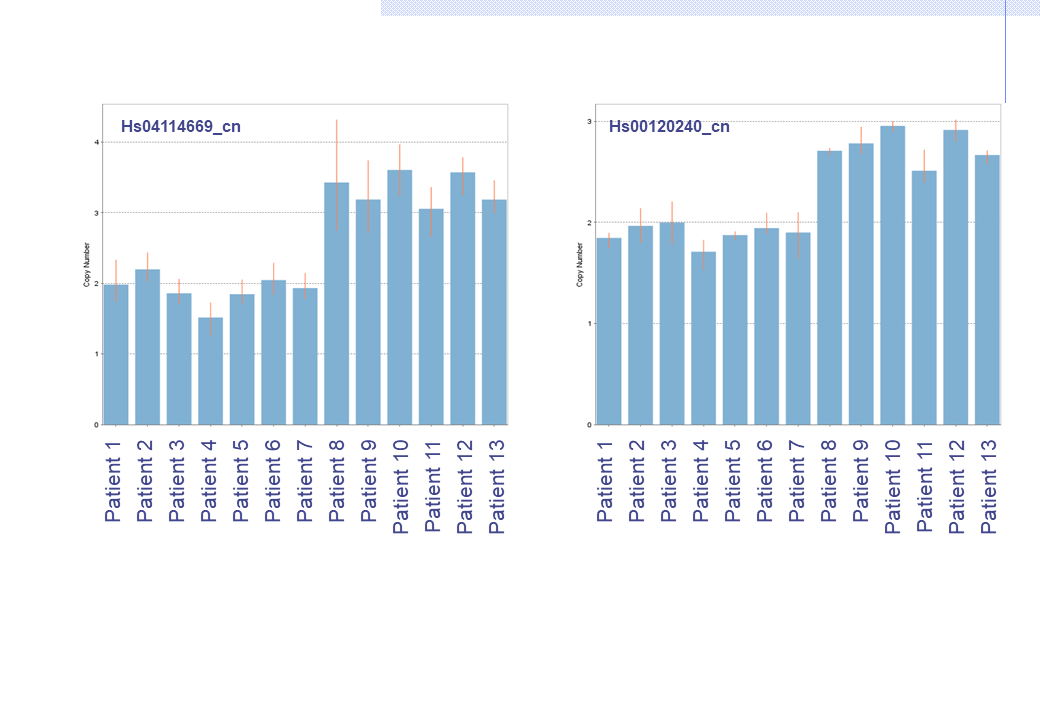


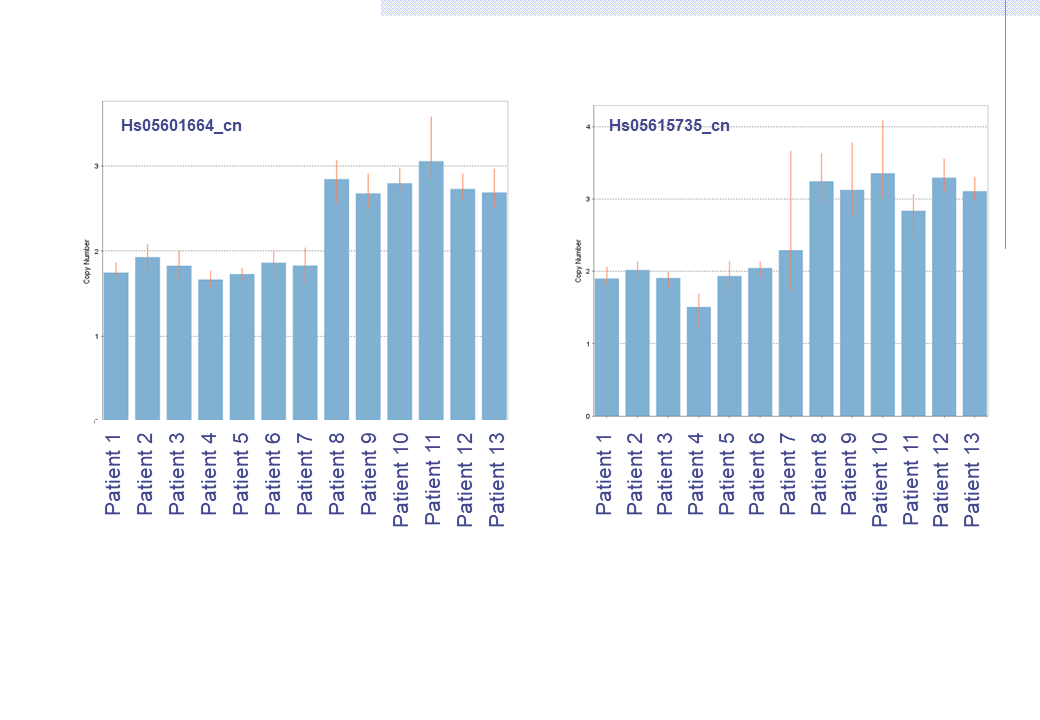


We validated 47,XXY in Patients 1–7 and 47,XXX in Patients 8-13 using TaqMan Copy Number Assays. The assays were performed using four probes targeting different regions of the X chromosome (Hs04114669_cn, Hs00120240_cn, Hs05601664_cn, and Hs05615735_cn). Bars indicate copy numbers of X chromosome predicted by TaqMan copy number assays. The results show copy number of two in patients with 47,XXY and copy number of three in patients with 47,XXX.
